# Integrated *Bacillus subtilis* Pretreatment, *Chlorella vulgaris* Cultivation, and *Trichoderma viride* Bioflocculation for Enhanced Municipal Wastewater Remediation and Biodiesel Production

**DOI:** 10.3390/molecules31081347

**Published:** 2026-04-20

**Authors:** Hongzhi Chen, Xiuren Zhou, Guifang Xu

**Affiliations:** 1 Department of Bioengineering, Xinxiang Institute of Engineering, 777# Xinfei Avenue South Section, Xinxiang 453700, China; chenhongzhi@xxgc.edu.cn; 2 Engineering Technology Research Center for the Development and High Value Utilization of Medicinal and Edible Plant Resources in the South Taihang Mountains of Xinxiang City, 777# Xinfei Avenue South Section, Xinxiang 453700, China; 3 School of Life Sciences, Henan Institute of Science and Technology, Huanlan Road, 655#, Xinxiang 453002, China; xgf@hist.edu.cn

**Keywords:** municipal wastewater valorization, *Chlorella vulgaris* cultivation, *Bacillus subtilis* pretreatment, fungal bioflocculation, microalgal biodiesel production, multi-microbial biorefinery, biomass resource conversion, circular bioeconomy

## Abstract

Municipal wastewater represents an underutilized secondary biomass resource rich in organic carbon and nutrients that can be valorized through biotechnological conversion. In this study, we developed an integrated multi-microbial biorefinery platform to transform municipal wastewater into value-added biofuel via sequential bacterial treatment, microalgal biomass generation, and fungal-assisted harvesting. Wastewater was first pretreated with *Bacillus subtilis* to enzymatically hydrolyze complex organic substrates and enrich the medium with bioactive metabolites, including auxins and gibberellins. The conditioned wastewater was subsequently used to cultivate *Chlorella vulgaris*, followed by biomass recovery using *Trichoderma viride* pellets as a sustainable bioflocculant. The integrated consortium significantly enhanced nutrient removal efficiency and promoted algal biomass accumulation, lipid enrichment, and biodiesel productivity compared to monoculture controls. Elevated hydrolytic enzyme activities (cellulase, protease, and amylases) facilitated organic matter conversion into bioavailable substrates, while increased phytohormone levels stimulated algal growth and lipid biosynthesis. Additionally, fungal bioflocculation substantially improved biomass recovery efficiency, reducing the need for energy-intensive harvesting technologies. This work highlights the potential of a biotechnology-driven approach for integrating wastewater remediation with biofuel production. By integrating microbial metabolism, enzymatic transformation, and sustainable separation processes, the proposed biorefinery system suggests a potentially low-carbon approach for simultaneous environmental remediation and biomass valorization, although further life cycle and energy balance analyses are required to validate this aspect.

## 1. Introduction

The rapid growth of the global population and accelerating urbanization have intensified the challenge of managing municipal wastewater effectively, making sustainable treatment strategies a priority in modern cities [1]. Beyond being an environmental burden, municipal wastewater contains substantial amounts of organic carbon, nitrogen, phosphorus, and trace nutrients that can be regarded as a secondary biological resource. Traditional wastewater remediation approaches primarily focus on contaminant removal rather than resource recovery. These methods often require high energy input or generate secondary pollution, limiting their sustainability [2,3,4]. However, physical techniques often fail to eliminate fine particulates and dissolved organics while requiring substantial energy input [5]; chemical methods risk secondary pollution and high operational costs [6]; biofilms demand precise environmental conditions and expensive media [7]; and membrane technologies involve significant maintenance expenses [8]. In this context, microalgae-based systems, particularly those employing *Chlorella* species, have emerged not only as eco-friendly and low-carbon treatment alternatives, but also as promising platforms for converting wastewater-derived nutrients into value-added biomass suitable for biodiesel production, thereby coupling environmental remediation with bioresource valorization [9,10].

A major bottleneck in microalgae-driven wastewater treatment and biofuel pathways is efficient biomass harvesting. Due to the small cell size and low settling rate of *Chlorella*, conventional methods like filtration or centrifugation are energy-intensive and costly [11,12]. Chemical flocculation, magnetic separation, and flotation can improve recovery, but they often increase cost or introduce contaminants [13,14]. Bioflocculation stands out as a promising, low-energy, environmentally benign option, where microbial agents (bacteria, fungi, or other microalgae) induce aggregation and settling [15]. Filamentous fungi, including *Aspergillus* and *Penicillium* species, have shown variable efficacy as bioflocculants, with flocculation performance depending on the fungal strain [16,17]. This variability underscores the need for further exploration of compatible, high-efficiency fungal partners that minimize ecological risks.

Wastewater composition strongly influences microalgal performance. Nutrient availability (e.g., nitrogen, phosphorus, and simple carbohydrates) and bioactive compounds both regulate growth and lipid accumulation [18,19]. Plant growth regulators, including auxins and gibberellins, are known to enhance microalgal development and biodiesel yields [20]. Certain *Bacillus* species, including *B. subtilis* and *Bacillus licheniformis*, are widely applied in bioremediation due to their production of diverse hydrolytic enzymes that degrade complex organics into bioavailable forms for algae, as well as secretion of phytohormones that support growth [21,22,23]. While some bacterial-algal interactions have been documented, for instance, *Azospirillum brasilense* promoting *Scenedesmus obliquus* via auxin production [24], the specific effects of *B. subtilis*-pretreated wastewater on *Chlorella vulgaris* remain relatively less explored, particularly in the context of integrated multi-microbial consortia involving sequential bacterial pretreatment and downstream biomass recovery.

*Bacillus subtilis*, a versatile and environmentally safe bacterium commonly employed in agriculture and bioprocessing, secretes a broad array of hydrolytic enzymes, including cellulases, amylases, and proteases, which convert macromolecules into usable substrates [25,26]. In addition, it produces plant growth regulators that stimulate development in plants and potentially in microalgae [26,27]. It has also shown promise in various wastewater remediation contexts [28,29]. *T. viride*, an eco-friendly filamentous fungus with established safety in environmental applications, offers potential as a bioflocculant [30,31]. Previous studies on *Bacillus licheniformis*–*Chlorella* consortia show that compared to using algae alone, their nutrient and COD removal rates are improved [32,33], and the consortium significantly increases biodiesel production of *Chlorella* [34]. However, the effects and potential mechanisms of the *B. subtilis*–*C. vulgaris*–*T. viride* consortium on the production of biodiesel by *C. vulgaris* remain elusive.

However, despite these advances, two critical gaps remain insufficiently addressed. First, the specific effects of *B. subtilis*-pretreated municipal wastewater on the growth performance, lipid accumulation, and biodiesel productivity of *Chlorella vulgaris* are still poorly understood. Second, existing studies have largely focused on single or dual microbial systems, whereas integrated multi-microbial strategies that simultaneously optimize wastewater remediation, biomass production, and harvesting efficiency remain underexplored. Therefore, developing a coordinated multi-microbial consortium that links these processes into a unified biorefinery framework represents an important yet unresolved challenge.

We hypothesized that pretreatment of municipal wastewater with *B. subtilis* could simultaneously enhance substrate bioavailability and enrich phytohormone levels, thereby promoting the growth, biomass accumulation, and lipid production of *C. vulgaris*. Furthermore, the incorporation of Trichoderma viride as a bioflocculant was expected to improve biomass harvesting efficiency, enabling a fully integrated and sustainable bioprocess.

Compared with previous studies focusing on single or dual microbial systems, this work provides a novel framework that integrates bacterial pretreatment, microalgal biomass generation, and fungal-assisted harvesting into a coordinated multi-microbial biorefinery platform. This integrated strategy not only improves pollutant removal efficiency but also enhances biomass productivity, lipid accumulation, and harvesting performance within a single system. Therefore, this study contributes to the development of sustainable wastewater valorization technologies by demonstrating a feasible approach for coupling environmental remediation with biofuel production in a low-carbon and resource-efficient manner.

## 2. Results

### 2.1. Pollutant Removal Efficiency in Municipal Wastewater Under Different Treatments

**Figure 1:**
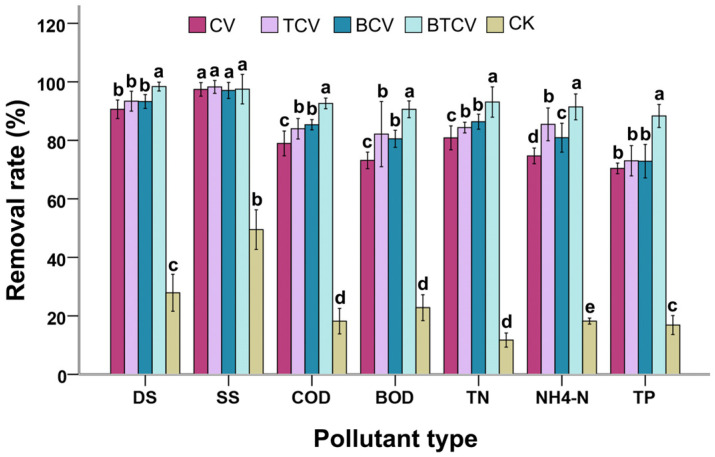
Effects of different treatments on pollutant removal efficiencies in municipal wastewater. CV: *Chlorella vulgaris* cultivated directly in untreated municipal wastewater. TCV: *C. vulgaris* cultured in municipal wastewater for 8 days, followed by bioflocculation using *Trichoderma viride*. BCV: Municipal wastewater pre-treated with *Bacillus subtilis* for 36 h prior to use as the cultivation medium for *C. vulgaris*, BTCV: *C. vulgaris* grown in municipal wastewater pre-treated with *B. subtilis*, followed by bioflocculation with *T. viride*. CK: Negative control (municipal wastewater receiving no treatment). DS: Dissolved solids. SS: Suspended solids. COD: Chemical oxygen demand. BOD: Biological oxygen demand. TN: Total nitrogen. NH_4_^+^-N: Ammonia nitrogen. TP: Total phosphorus. Different letters (a–e) indicate significant differences among treatments (*p* < 0.05).

Figure 1 illustrates the removal percentages of key wastewater parameters across treatments. The BTCV consortium (*B. subtilis* pretreatment + *C. vulgaris* cultivation + *T. viride* bioflocculation) achieved the highest removal efficiencies for dissolved solids (DS), biochemical oxygen demand (BOD), chemical oxygen demand (COD), ammonia nitrogen (NH_4_^+^-N), total nitrogen (TN), and total phosphorus (TP). These values were markedly superior to those in all other groups (e.g., COD removal: BTCV vs. CV, *p* = 0.00; BTCV vs. BCV, *p* = 0.00). Removal of suspended solids (SS) showed no significant differences among CV, TCV, BCV, and BTCV, indicating that solid particulates were comparably reduced regardless of pretreatment or flocculation method.

Specifically, BTCV exhibited significantly higher removal efficiencies for COD, BOD, TN, and NH compared to CV, TCV, and BCV (*p* < 0.03 in all comparisons), indicating that the integrated application of bacterial pretreatment, algal culture, and fungal flocculation generated synergistic effects that exceeded those achieved by individual treatments or any pairwise integration. Overall, the full three-organism consortia (BTCV) is consistent with synergistic pollutant degradation beyond that observed in pairwise consortia or algal monoculture, reflecting complementary roles among *B. subtilis*, *C. vulgaris*, and *T. viride*.

### 2.2. Impact of B. subtilis Pretreatment on C. vulgaris Growth Parameters

**Figure 2:**
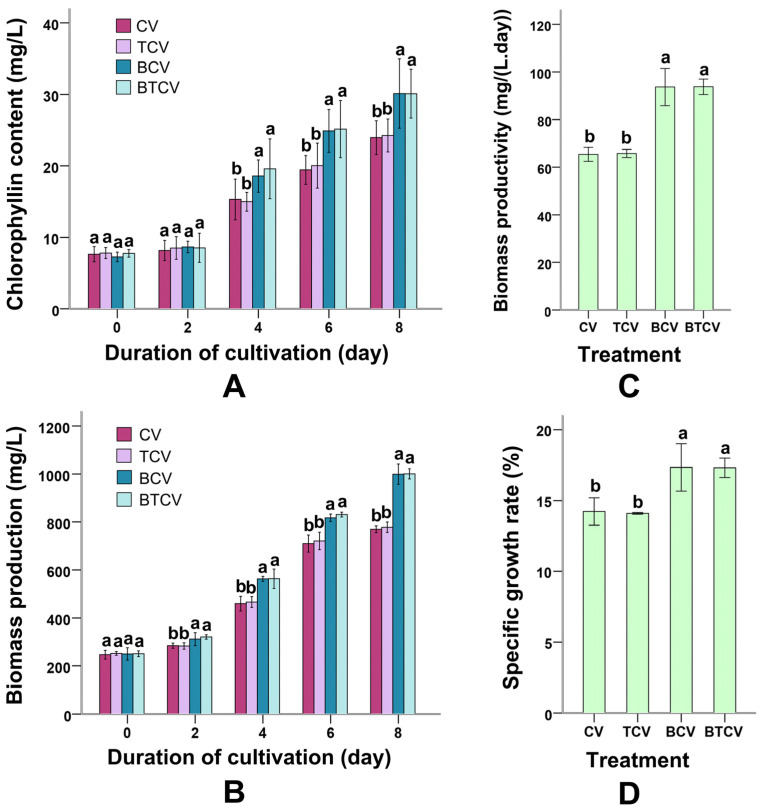
Impact of different treatments on chlorophyll concentration, biomass yield, biomass productivity, and specific growth rate of *Chlorella vulgaris*. (**A**) Chlorophyll content under various treatment conditions. (**B**) Biomass yield under various treatment conditions. (**C**) Biomass productivity under various treatment conditions. (**D**) Specific growth rate under various treatment conditions. CV: *C. vulgaris* cultivated directly in municipal wastewater as the growth medium. TCV: *C. vulgaris* grown in municipal wastewater for 8 days, followed by bioflocculation with *Trichoderma viride*. BCV: Municipal wastewater pretreated with *Bacillus subtilis* for 36 h before being used to culture *C. vulgaris*. BTCV: C. vulgaris cultivated in municipal wastewater pretreated with *B. subtilis*, and subsequently bioflocculated with *T. viride*. Different letters (a, b) indicate significant differences among treatments (*p* < 0.05).

Figure 2 summarizes chlorophyll content, biomass accumulation, biomass productivity, and specific growth rate in *C. vulgaris* across treatments. Cultures grown in *B. subtilis*-pretreated wastewater (MWTB; groups BCV and BTCV) exhibited significantly elevated chlorophyll levels starting from day 4 compared to those in untreated municipal wastewater (CV and TCV) (Figure 2A). For instance, at day 4, chlorophyll content in BTCV was higher (*p* = 0.01) than in CV. Biomass production (316.57 ± 35 mg/L) in MWTB-based cultures diverged positively from day 2 onward, remaining higher throughout the 8-day period (Figure 2B).

Both biomass productivity and specific growth rate followed the same pattern, with MWTB cultures showing statistically superior performance relative to untreated controls (*p* = 0.00) (Figure 2C,D). Notably, the divergence in biomass accumulation between pretreated (BCV, BTCV) and untreated groups (CV, TCV) became evident as early as day 2, suggesting that bacterial pretreatment rapidly modifies the growth environment and accelerates algal proliferation.

### 2.3. Biodiesel Yield, Productivity, and Lipid Content in C. vulgaris

**Figure 3:**
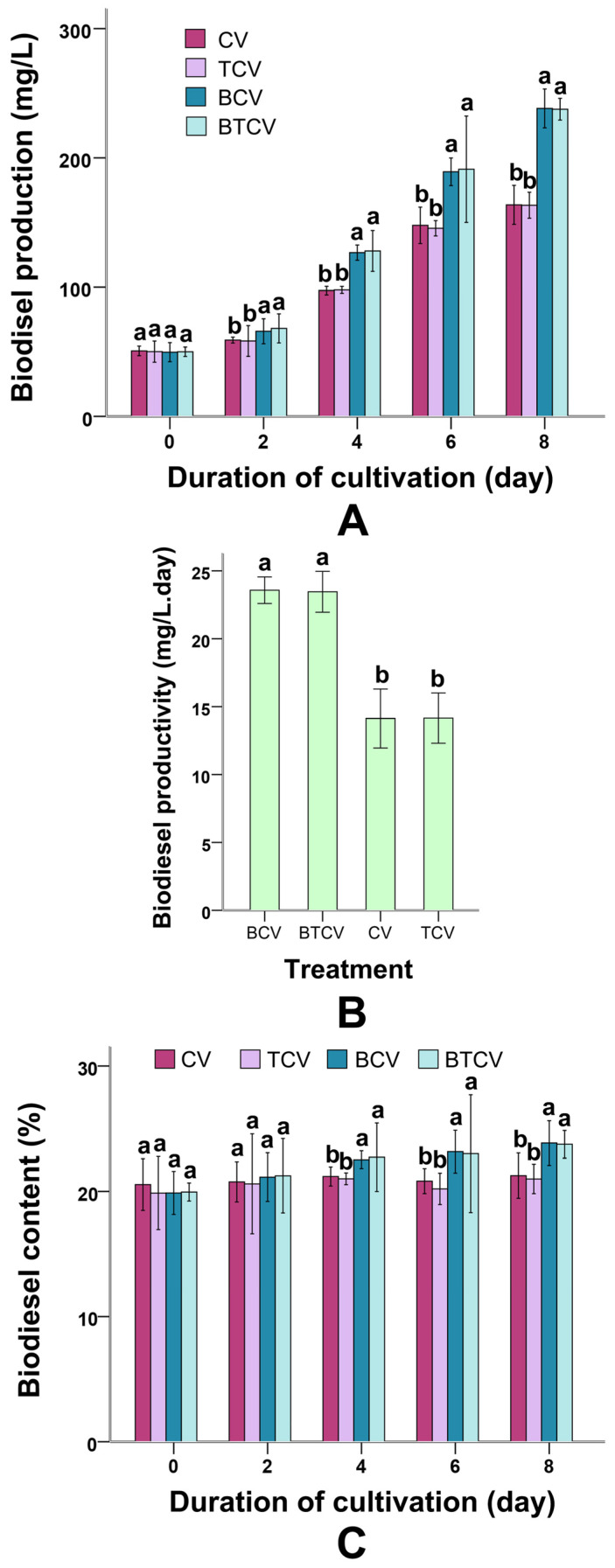
Influence of various treatments on biodiesel production performance of *Chlorella vulgaris*. (**A**) Biodiesel yield under different treatment conditions. (**B**) Biodiesel productivity under different treatment conditions. (**C**) Biodiesel content under different treatment conditions. CV: *C. vulgaris* cultivated directly in municipal wastewater. TCV: *C. vulgaris* grown in municipal wastewater for 8 days followed by bioflocculation using *Trichoderma viride*. BCV: Municipal wastewater pretreated with *Bacillus subtilis* for 36 h prior to inoculation of *C. vulgaris*. BTCV: C. vulgaris cultivated in municipal wastewater pretreated with *B. subtilis*, followed by bioflocculation with *T. viride*. No biodiesel was present in the municipal wastewater at this stage; values reflect baseline measurements prior to lipid accumulation. Different letters (a, b) indicate significant differences among treatments (*p* < 0.05).

Biodiesel-related parameters are presented in Figure 3. At time 0, biodiesel content was negligible across all treatments, confirming that lipid accumulation occurred progressively during algal cultivation rather than being present in the initial wastewater. In MWTB-based cultures (BCV and BTCV), biodiesel production became significantly higher (*p* < 0.01 in all comparisons after day 4) than in untreated wastewater cultures (CV and TCV) after day 4 (Figure 3A). For example, at day 6, biodiesel yield in BTCV exceeded that in CV (154.62 ± 23 mg/L, *p* = 0.00). Biodiesel productivity was consistently elevated in the pretreated groups across the cultivation period (*p* = 0.00) (Figure 3B).

Lipid (oil) content (24.13 ± 4.68%) in *C. vulgaris* also increased substantially under MWTB conditions, with statistically significant differences (*p* = 0.02) emerging from day 4 and persisting thereafter (Figure 3C). The simultaneous increase in both biomass and lipid content suggests that the enhanced biodiesel production in MWTB-based cultures was not solely due to biomass accumulation but also to improved lipid biosynthesis efficiency.

### 2.4. Hydrolytic Enzyme Activities in B. subtilis-Pretreated Wastewater

**Figure 4:**
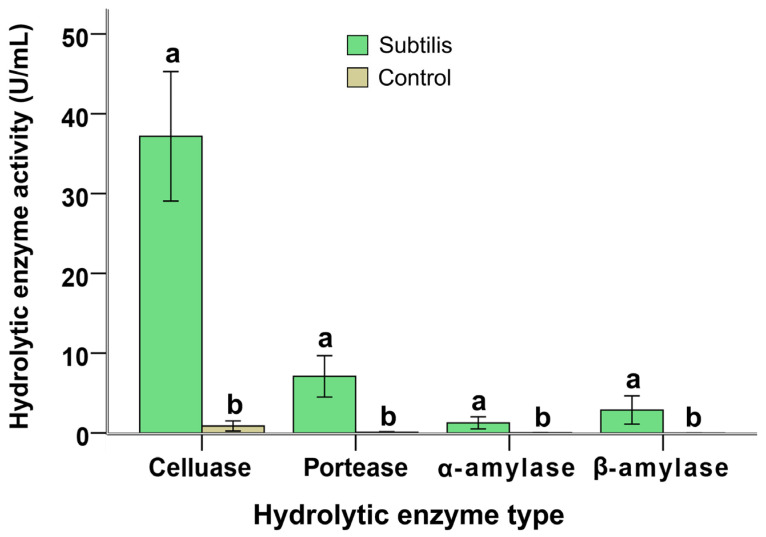
Activities of key hydrolytic enzymes, including cellulase, neutral protease, α-amylase, and β-amylase, under different treatment conditions. The figure illustrates the activity levels of various hydrolytic enzymes in wastewater following a 36 h treatment with *B. subtilis*. “Subtilis” refers to municipal wastewater subjected to 36 h incubation with *B. subtilis*, whereas “Control” denotes untreated municipal wastewater without any intervention. All enzyme names have been standardized to conventional biochemical nomenclature. Different letters (a, b) indicate significant differences among treatments (*p* < 0.05).

The activities of cellulase, neutral protease, α-amylase, and β-amylase were significantly enhanced in the bacterial pretreatment groups (Figure 4). Compared to untreated municipal wastewater (control) (0.85 ± 0.12 U/mL), MWTB displayed markedly higher cellulase activity (37.17 ± 4.06 U/mL, *p* = 0.00). Protease activity was also significantly increased (7.09 ± 1.30 U/mL). In addition, detectable levels of α-amylase and β-amylase were present in MWTB but were negligible in the control (*p* = 0.00). These elevated hydrolytic activities are associated with the capacity of *B. subtilis* to break down complex macromolecules, including cellulose, proteins, and starches, into simpler and more bioavailable compounds such as sugars and amino acids.

### 2.5. Plant Growth Regulator Concentrations in Pretreated Wastewater

**Figure 5:**
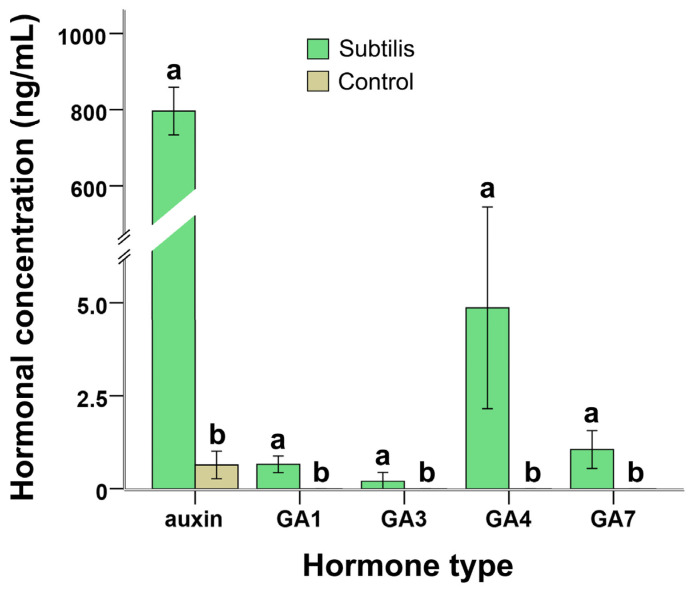
Effect of *Bacillus subtilis* application on the levels of plant growth regulators in municipal wastewater. “Subtilis” represents municipal wastewater treated with *B. subtilis* for 36 h, while “Control” refers to wastewater that received no treatment. GA denotes gibberellin. Different letters (a, b) indicate significant differences among treatments (*p* < 0.05).

Figure 5 compares phytohormone levels in MWTB and untreated wastewater. Auxin concentration reached 796.38 ± 31.28 ng/L in MWTB, representing a substantial increase over the trace level (0.64 ± 0.19 ng/L) in the control. Gibberellins (GA_1_, GA_3_, GA_4_, GA_7_) were detectable in MWTB, with GA_4_ attaining 4.86 ± 1.35 ng/L, whereas none were measurable in untreated wastewater. This enrichment is correlated with the observed enhancement of *C. vulgaris* growth and lipid accumulation; however, direct causal relationships were not established in this study.

Compared with other phytohormones, GA_4_ exhibited relatively higher variability among replicates, as indicated by the larger error bars. This may reflect the dynamic and sensitive nature of gibberellin biosynthesis during microbial pretreatment.

### 2.6. Flocculation Efficiency of C. vulgaris Biomass

**Figure 6:**
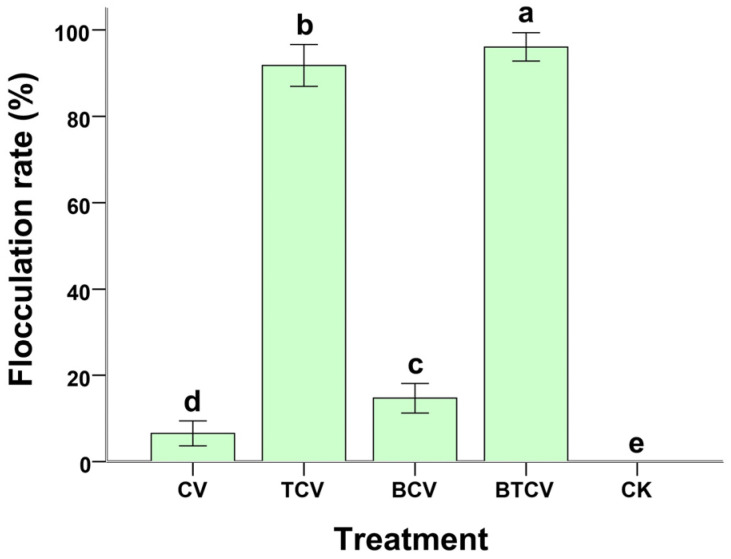
Influence of various treatments on the flocculation efficiency of *Chlorella vulgaris*. CV: *C. vulgaris* cultivated directly in municipal wastewater. TCV: *C. vulgaris* grown in municipal wastewater for 8 days, followed by bioflocculation using *Trichoderma viride*. BCV: Municipal wastewater pretreated with *Bacillus subtilis* for 36 h prior to its use as the culture medium for *C. vulgaris*. BTCV: *C. vulgaris* cultured in municipal wastewater pretreated with *B. subtilis*, and subsequently subjected to bioflocculation with *T. viride*. Different letters (a–e) indicate significant differences among treatments (*p* < 0.05).

Flocculation performance is depicted in Figure 6. Bioflocculation using *T. viride* pellets yielded significantly higher recovery rates in BTCV than in TCV (91.35 ± 3.46%, *p* = 0.00). Even in self-flocculation mode, BCV showed markedly better settling efficiency than CV (6.85 ± 2.38%, *p* = 0.00). These differences support the possibility that *B. subtilis* pretreatment enhances algal cell properties or surface characteristics, thereby facilitating both self- and bioflocculation processes.

### 2.7. pH Dynamics in Wastewater During Treatment

**Figure 7:**
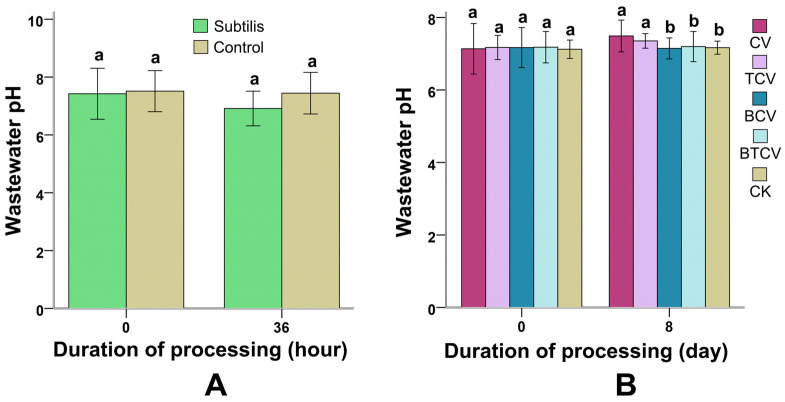
Variation in pH under different treatment conditions during cultivation. (**A**) Effect of *B. subtilis* pretreatment on wastewater pH. Panel (**A**) presents the pH variation during the 36 h incubation of municipal wastewater with *B. subtilis*. (**B**) pH variation of municipal wastewater under different treatment conditions. CV: *C. vulgaris* cultivated directly in municipal wastewater. TCV: *C. vulgaris* grown in municipal wastewater for 8 days and subsequently bioflocculated with *Trichoderma viride*. BCV: Municipal wastewater pretreated with *B. subtilis* for 36 h prior to use as the culture medium for *C. vulgaris*. BTCV: *C. vulgaris* cultured in municipal wastewater pretreated with *B. subtilis*, followed by bioflocculation with *T. viride*. CK: Untreated municipal wastewater serving as the negative control. Subtilis denotes municipal wastewater subjected to 36 h treatment with *B. subtilis*, whereas Control refers to wastewater without any treatment. Different letters (a, b) indicate significant differences among treatments (*p* < 0.05).

pH changes are illustrated in Figure 7. During the 36 h *B. subtilis* pretreatment, wastewater pH remained stable and comparable to the untreated control (*p* = 0.26) (Figure 7A). After 8 days of *C. vulgaris* cultivation, however, distinct patterns emerged (Figure 7B). Groups CV and TCV (untreated wastewater) exhibited significant pH increases relative to the control (CK) (*p* = 0.00 to 0.01). In contrast, BCV and BTCV (MWTB-based) maintained pH values close to the initial and control levels (*p* = 0.963 to 1.00). These findings are in line with *B. subtilis* pretreatment being able to alleviate the alkalizing effect frequently observed during *C. vulgaris* growth in raw municipal wastewater.

The observed pH variation reflects the metabolic activity of the microbial system. During bacterial pretreatment, organic matter degradation and metabolite production may alter the pH, which in turn affects nutrient solubility and enzyme activity. Such changes create a more favorable environment for subsequent microalgal growth.

## 3. Discussion

### 3.1. Synergistic Pollutant Removal Through Multi-Microbial Interactions

Municipal wastewater remediation using *C. vulgaris* alone achieves substantial reductions in COD, BOD, TN, and TP, as previously documented [35,36,37]. Bacterial species, including various *Bacillus* strains, have independently indicated pollutant degradation capabilities [38,39]. While these studies primarily frame wastewater treatment as a remediation strategy, the organic carbon and nutrients present in municipal wastewater also represent a convertible biological resource.

The present study extends these observations by showing that sequential integration of *B. subtilis* pretreatment with *C. vulgaris* cultivation, followed by *T. viride*-assisted harvesting (BTCV), yields consistently superior removal efficiencies across most parameters compared to algal monoculture (CV) or pairwise consortia (TCV, BCV). Beyond pollutant removal, this system redirects wastewater-derived carbon and nutrients into microalgal biomass. This process effectively couples remediation with biomass production.

This enhanced performance likely arises from complementary metabolic activities: *B. subtilis* initiates breakdown of complex organics (evident in elevated hydrolytic enzyme levels in MWTB; Figure 4), while *C. vulgaris* assimilates released nutrients and photosynthetically fixes carbon, and *T. viride* contributes to final biomass aggregation and residual pollutant capture. The additive or synergistic effects align with emerging evidence that bacteria–algae consortia outperform single-species systems in wastewater bioremediation [40,41,42]. The incorporation of a fungal bioflocculant appears to further differentiate this strategy, indicating that multi-kingdom microbial partnerships could potentially improve both degradation efficiency and downstream separation, which represents a critical step in resource recovery systems.

The synergy between *B. subtilis* and *C. vulgaris* can be interpreted as a two-tier interaction involving both substrate transformation and metabolic stimulation. Specifically, bacterial hydrolysis of complex organic matter increases the pool of low-molecular-weight compounds, while simultaneously produced phytohormones act as signaling molecules that regulate algal cell division and metabolic activity. This coordinated effect enables a more efficient coupling between nutrient availability and cellular assimilation processes.

### 3.2. B. subtilis Pretreatment Promotes C. vulgaris Growth and Biomass Yield

Pretreatment with *B. subtilis* markedly enhanced chlorophyll content, biomass accumulation, productivity, and specific growth rate of *C. vulgaris* in MWTB-based cultures (Figure 2). These improvements may be associated with two primary factors.

First, *B. subtilis* secreted substantial amounts of auxins and gibberellins into the medium (Figure 5), compounds known to stimulate microalgal cell division, pigment synthesis, and overall development [20]. Supplementary experiments with exogenous phytohormones showed an association with positive effects on *C. vulgaris* biomass in municipal wastewater (Figure S1A), which is consistent with the hypothesis that bacterial-derived regulators may contribute to the observed growth promotion, similar to auxin-mediated effects reported for *Azospirillum brasilense* on *Scenedesmus obliquus* [24].

Second, the elevated activities of cellulase, protease, and amylases indicate that macromolecular organic compounds in wastewater were actively converted into soluble and bioavailable forms, such as monosaccharides, oligosaccharides, and free amino acids. These smaller molecules are more readily transported across algal cell membranes, thereby enhancing nutrient uptake efficiency and supporting rapid biomass accumulation. This nutrient enrichment may resemble the growth-promoting effect typically seen with glucose supplementation in *Chlorella* cultures [43,44]. Together, these enzymatic and hormonal contributions create an optimized nutritional and signaling environment that accelerates algal proliferation beyond what untreated municipal wastewater can provide.

### 3.3. Enhanced Biodiesel Yield and Lipid Accumulation via Bacterial Conditioning

*B. subtilis* pretreatment not only increased overall biodiesel production and productivity but also elevated intracellular lipid content in *C. vulgaris* (Figure 3). This dual benefit is consistent with the biomass increase described above and with literature showing that phytohormones such as IAA and GA, together with carbon-rich substrates, promote lipid biosynthesis in microalgae [45,46,47].

Elevated auxin and gibberellin levels in MWTB, together with the release of simple sugars from bacterial hydrolysis, likely stimulated lipid accumulation pathways. Exogenous regulator addition experiments further validated this link, showing significant rises in biodiesel yield and content (Figure S1B,C). Unlike stress-induced lipid enhancement [48], the *B. subtilis*-mediated conditioning appears to support high biomass without compromising growth, enabling simultaneous productivity and lipid enrichment. From a resource valorization standpoint, this coordinated enhancement is associated with the rechanneling of wastewater-derived carbon, beyond mere removal, into energy-dense storage compounds suitable for downstream biofuel applications. These findings hint that bacterial pretreatment might help influence nutrient availability and signaling pathways in microalgae, potentially offering a complementary approach to support greater biomass accumulation and lipid biosynthesis.

Taken together, these results demonstrate that the observed improvements in algal growth and biodiesel production are closely linked to bacterial-mediated changes in nutrient composition and signaling environment, highlighting the importance of microbial synergy in wastewater-based bioprocesses.

### 3.4. Improved Harvesting Efficiency Through B. subtilis Conditioning and T. viride Bioflocculation

Harvesting remains a critical bottleneck in microalgal biotechnology. Our results show that *B. subtilis* pretreatment significantly improves both self-flocculation and *T. viride*-mediated bioflocculation of *C. vulgaris* (Figure 6). The higher recovery rates in BCV and BTCV suggest that bacterial modification of wastewater chemistry may alter algal cell surface properties. This effect may result from changes in extracellular polymeric substances, surface charge, or nutrient-induced physiological shifts, which promote cell aggregation [15,49,50,51,52,53].

The introduction of *T. viride* pellets as a bioflocculant represents a key innovation, achieving particularly high flocculation in BTCV. This fungal and algal interaction, combined with prior bacterial conditioning, may provide a low-energy and eco-friendly harvesting pathway that reduces dependence on chemical flocculants or energy-intensive centrifugation. By integrating biomass production and separation within the same biological cascade, the system strengthens the feasibility of wastewater-based bioresource platforms.

It is also important to note that all experiments were conducted in biological triplicates, and the relatively small standard deviations observed across treatments (Figure 2, Figure 3, Figure 4, Figure 5 and Figure 6) indicate good reproducibility of the consortia. However, some variability was observed in enzyme activity and phytohormone measurements, which may be attributed to the inherent heterogeneity of municipal wastewater composition. Future studies should further investigate the stability of the consortia under fluctuating environmental conditions.

### 3.5. pH Stabilization During Combined Cultivation

*C. vulgaris* cultivation in untreated wastewater typically elevates pH due to CO_2_ consumption and metabolic byproducts [54]. This alkalization was evident in the CV and TCV groups (Figure 7B). However, pH remained stable in BCV and BTCV, closely matching the control. *B. subtilis* is known to produce organic acids (e.g., lactic and malic acids) during metabolism [55,56], which likely buffered the medium against algal-induced pH rise. This stabilization maintains a more consistent biochemical environment, which may indirectly support sustained biomass productivity and lipid accumulation during prolonged cultivation.

pH is a key environmental factor influencing enzyme activity, nutrient availability, and microalgal physiology. The observed pH trends suggest that bacterial pretreatment may help stabilize or optimize the culture environment, thereby indirectly contributing to enhanced algal growth and lipid production.

### 3.6. Prospects for Multi-Microbial, Low-Carbon Wastewater Biorefinery Consortia

The integration of *B. subtilis* pretreatment, *C. vulgaris* cultivation, and *T. viride* bioflocculation offers a coordinated microbial platform that links pollutant removal, biomass production, and energy-oriented product generation within a single operational framework. Rather than functioning solely as a treatment chain, the consortium resembles a biologically structured cascade in which pretreatment (enzymatic hydrolysis), conversion (algal biomass and lipid synthesis), and separation (fungal bioflocculation) occur sequentially.

Such functional stratification parallels the conceptual stages of a biorefinery process, yet is achieved through natural microbial interactions without genetic modification or intensive chemical input. By converting wastewater-derived organic matter into lipid-rich biomass suitable for biodiesel production, the consortia align with the potential of municipal wastewater to serve as a secondary feedstock for renewable energy generation within a circular urban metabolism.

Future work should systematically compare additional bacterial and fungal partners to identify optimal consortia. Deeper mechanistic insights, via metabolomics, transcriptomics, and proteomics, will elucidate how bacterial metabolites and fungal interactions modulate algal physiology and lipid pathways. Scaling studies and life cycle assessments will be essential to evaluate the environmental footprint and techno-economic feasibility. Ultimately, such integrated multi-kingdom microbial consortia hold strong potential for advancing circular, low-carbon water–energy nexus strategies.

It should be emphasized that the present study does not establish direct causal relationships between specific bacterial metabolites (e.g., enzymes or phytohormones) and algal physiological responses. Future studies employing controlled supplementation, inhibitor-based approaches, or omics-level analyses are required to validate these proposed mechanisms.

## 4. Materials and Methods

### 4.1. Preparation of Inoculum Stocks for B. subtilis and C. vulgaris

Pre-cultured *B. subtilis* suspension (used as inoculum) was freshly prepared prior to wastewater inoculation. *B. subtilis* (strain SHBCC D50761, Shanghai Bioresource Collection Center, Shanghai, China), 5 mg of lyophilized powder, was activated in 10 mL LB broth at 37 °C and 200 rpm for 12 h. A 200 µL aliquot was transferred to fresh 10 mL LB for secondary activation under identical conditions. Activated cells were streaked on antibiotic-free LB agar and incubated at 37 °C for 12 h. A single colony was inoculated into LB broth and shaken at 220 rpm and 37 °C for 12 h, then scaled up by adding 500 µL to 500 mL LB in a 1 L flask until reaching 1 × 10^9^ CFU/mL (monitored via OD_600_ on a Shimadzu UV-1800 spectrophotometer (Shimadzu Corporation, Kyoto, Japan), with concentration estimated from a pre-established standard curve).

Exponentially growing algal cultures used for inoculation were prepared prior to inoculation. Five mg lyophilized powder of *C. vulgaris* (strain FACHB-8, Freshwater Algae Culture Collection at the Institute of Hydrobiology, Wuhan, China) was resuspended in 200 mL autoclaved BG11 medium in 500 mL flasks equipped with aeration. Cultures were incubated at 25 °C under 7000 lx with a 14:10 light–dark cycle for 48 h. A 50 mL aliquot was then transferred to 500 mL fresh BG11 in 1 L flasks under the same conditions. The algal stock culture was established by growing *Chlorella vulgaris* until the cell density reached approximately 1 × 10^8^ cells mL^−1^ (concentration calculated from a calibrated OD-cell number curve). This culture was subsequently used as the inoculum source for introducing microalgae into municipal wastewater systems.

### 4.2. Preparation of T. viride Pellets as Bioflocculant

*T. viride* mycelium (strain bio-74884, Beijing Baiou Bowei Biotechnology Co., Ltd., Beijing, China) was transferred from slant culture to PDA agar plates and incubated at 28 °C for 48 h. Mycelial fragments and spores were inoculated into 200 mL PDA broth in 500 mL flasks and shaken at 150 rpm and 28 °C for 36 h. Pellets (3.0–6.0 mm diameter) formed at this stage were used directly as the bioflocculant for *C. vulgaris* harvesting.

### 4.3. Experimental Design and Cultivation Conditions

The study comprised four treatment groups and one untreated control, as summarized in Table 1.

Fresh untreated municipal wastewater was collected from the Yuanyang Wastewater Treatment Plant, Xinxiang City, Henan Province. For BCV and BTCV groups, 450 mL of wastewater in 1 L flasks was inoculated with 50 mL *B. subtilis* stock and incubated at 37 °C and 220 rpm with 2.50 L/min aeration for 36 h. Subsequently, 50 mL *C. vulgaris* stock was added to the pretreated wastewater (MWTB) and cultivated for 8 days at 7000 lx, 14:10 light–dark cycle, 25 °C, and 2.50 L/min aeration. CV and TCV used untreated MW under identical algal cultivation conditions. For TCV and BTCV, post-cultivation cultures were heat-treated (100 °C, 3 min), mixed with *T. viride* pellet broth at 1:2.5 (*v*/*v*), and flocculated for 48 h. Self-flocculation (SF) was applied to CV and BCV after heat treatment. All groups were run in triplicate.

Prior to flocculation, cultures were subjected to heat treatment (100 °C for 3 min) to terminate microbial metabolic activity and standardize the physicochemical conditions during the flocculation process. This step was intended to prevent further biological transformations that could interfere with flocculation efficiency assessment. It should be noted that biomass used for lipid extraction and biodiesel analysis was collected prior to heat treatment to avoid potential thermal degradation of cellular components. Nevertheless, the possible effects of heat treatment on cell structure and aggregation behavior should be considered when interpreting flocculation results.

To minimize potential overestimation of algal biomass and lipid production, separate control experiments were conducted in which Bacillus subtilis was cultivated in municipal wastewater under identical conditions without *C*. *vulgaris*. The resulting bacterial biomass and lipid yield were quantified independently and used as baseline reference values. These values were applied as correction factors to estimate the net contribution of *C. vulgaris* in mixed systems. It should be noted that this approach provides an approximation and may not fully account for dynamic interactions between bacterial and algal metabolism in co-culture systems.

All sampling time points used in this study are explicitly defined and consistently applied across the experimental design and figure presentation. Time 0 was defined as the point immediately after inoculation of *C*. *vulgaris* into the cultivation medium (untreated municipal wastewater or *B. subtilis*-pretreated wastewater), following the completion of bacterial pretreatment where applicable.

### 4.4. Wastewater Parameter Analysis

Samples (30 mL) from treated and untreated wastewater were analyzed for suspended solids (SS), dissolved solids (DS), BOD, COD, TN, TP, NH_4_^+^-N, and pH following standard methods of the American Public Health Association [57].

### 4.5. Flocculation Rate Determination

The initial OD_680_ of the *C. vulgaris* culture was measured before flocculation. After flocculation, supernatant OD_680_ was recorded. Flocculation rate (%) was calculated as:

Flocculation rate = [(OD_initial − OD_final)/OD_initial] × 100

### 4.6. Chlorophyll Content Measurement

Chlorophyll extraction and quantification followed the procedure detailed in [34], with absorbance at 663.8 nm and 646.8 nm used to compute chlorophyll a, b, and total chlorophyll contents via equations from [58,59].

### 4.7. Biomass, Productivity, and Specific Growth Rate

Dry biomass was determined as described in [34,60]. Washed pellets (from 50 mL culture) were dried at 105 °C to constant weight. Biomass production (mg/L), productivity (mg/L/day), and specific growth rate (%/day) were calculated using the standard equations provided therein.

### 4.8. Biodiesel Production, Content, and Productivity

Lipid extraction, transesterification, and biodiesel quantification were performed following the modified protocol of [61], as outlined in [34]. Freeze-dried biomass was milled, lipids extracted with chloroform:methanol (2:1 *v*/*v*), transesterified with methanolic NaOH, purified, and dried. Biodiesel production (mg/L), content (%), and productivity (mg/L/day) were computed using the respective equations.

### 4.9. Hydrolytic Enzyme Activities and Phytohormone Quantification

Enzyme assays (cellulase via DNS method [62], protease via Folin-phenol [63], α- and β-amylase [64], lipase [65]) and plant growth regulator contents followed [66,67], with measurements conducted on pretreated wastewater samples.

### 4.10. Statistical Analysis

Data were checked for normality and homogeneity of variance. One-way ANOVA followed by Tukey’s HSD post hoc test was used to compare treatments, while independent *t*-tests were used to evaluate differences in enzymes and regulators. Significance was set at *p* < 0.05. Exact *p*-values are reported where applicable, and effect sizes were considered when interpreting differences among treatments. All data are presented as mean ± standard deviation (SD) based on three independent biological replicates. Analyses and figures were generated using SPSS 19.0 (SPSS Inc., Chicago, IL, USA).

## 5. Conclusions

This study suggests that the integration of *B. subtilis* pretreatment, *C. vulgaris* cultivation, and *T. viride*-assisted bioflocculation can enhance nutrient removal, algal biomass accumulation, lipid content, and harvesting efficiency in municipal wastewater systems. The observed improvements are associated with increased hydrolytic enzyme activities and elevated phytohormone levels in pretreated wastewater, which may contribute to enhanced substrate availability and algal growth. This study advances current knowledge by showing that multi-kingdom microbial integration may simultaneously address three key bottlenecks in wastewater-based biofuel systems: substrate availability, biomass productivity, and harvesting efficiency. However, the mechanistic relationships between bacterial metabolites and algal physiological responses remain to be further validated. In addition, the scalability and environmental performance of the consortium require further investigation through pilot-scale studies and life cycle assessment. Overall, this work may provide experimental evidence supporting the feasibility of multi-microbial integration for wastewater valorization, while highlighting key directions for future research.

## Figures and Tables

**Table 1:** Experimental treatments and control.

Treatment	CV	TCV	BTCV	BCV	CK
Medium	MW	MW	MWTB	MWTB	MW
Duration of cultivation (days)	8	8	8	8	8
Flocculation	SF	BFT	BFT	SF	SF

CV: *Chlorella vulgaris* cultured in municipal wastewater (MW) for 8 days, followed by self-flocculation (SF). TCV: *C. vulgaris* cultured in MW for 8 days, followed by bioflocculation with *Trichoderma viride* (BFT). BTCV: MW pretreated with *Bacillus subtilis* (MWTB) for 36 h, then used to culture *C. vulgaris* for 8 days, followed by BFT. BCV: MW pretreated with *B. subtilis* for 36 h, then used to culture *C. vulgaris* for 8 days, followed by SF. CK: Untreated MW control (no *B. subtilis* or *C. vulgaris*).

## Data Availability

The original contributions presented in this study are included in the article/Supplementary Material. Further inquiries can be directed to the corresponding author.

## Supplementary Materials

The following supporting information can be downloaded at https://www.mdpi.com/article/10.3390/molecules31081347/s1: Figure S1: Effects of exogenously applied plant growth regulators on the biomass production, biodiesel yield, and biodiesel content of Chlorella vulgaris. (A) Biomass production in response to exogenous plant growth regulator supplementation. (B) Biodiesel yield following exogenous plant growth regulator treatment. (C) Biodiesel content under exogenous plant growth regulator treatment. The final concentrations of plant growth regulators in municipal wastewater were adjusted to levels comparable to those of auxin and gibberellins detected after 36 h of Bacillus subtilis treatment. CV: Chlorella vulgaris cultured directly in municipal wastewater. PGR: Chlorella vulgaris cultured in municipal wastewater supplemented with exogenous plant growth regulators.; Table S1: Biomass and biodiesel content of Bacillus subtilis cultured for 8 days under conditions similar to those used for Chlorella growth. The cultivation duration was 0, 2, 4, 6, and 8 days. Biomass and biodiesel content are presented as mean ± standard deviation.

## Abbreviations

The following abbreviations are used in this manuscript:

BCV	Municipal wastewater pretreated with *Bacillus subtilis* for 36 h, then used to culture *Chlorella vulgaris* for 8 days, followed by self-flocculation
BOD	Biological oxygen demand
BTCV	Municipal wastewater pretreated with *Bacillus subtilis* for 36 h, then used to culture *Chlorella vulgaris* for 8 days, followed by bioflocculation with *Trichoderma viride*
CFU	Colony-forming unit
CK	Untreated municipal wastewater control (no *Bacillus subtilis* or *Chlorella vulgaris*)
COD	Chemical oxygen demand
CV	*Chlorella vulgaris* cultured in municipal wastewater for 8 days, followed by self-flocculation
DS	Dissolved solids
GA	Gibberellic acid
IAA	Indole-3-acetic acid
LB	Luria–Bertani medium
MW	Municipal wastewater
MWTB	Municipal wastewater pretreated with *Bacillus subtilis*
PDA	Potato dextrose agar
SS	Suspended solids
TCV	*Chlorella vulgaris* cultured in municipal wastewater for 8 days, followed by bioflocculation with *Trichoderma viride*
TN	Total nitrogen
TP	Total phosphorus
