# Supplementary material for: Integrated Bacillus subtilis Pretreatment, Chlorella vulgaris Cultivation, and Trichoderma viride Bioflocculation for Enhanced Municipal Wastewater Remediation and Biodiesel Production
Source: Molecules. 2026 Apr 20;31(8):1347. doi: 10.3390/molecules31081347 (PMC13119039; doi:10.3390/molecules31081347)
Supplement: Supplementary file 1 [file molecules-31-01347-s001.zip › molecules-4237723-supplementary.pdf]

Table S1. Biomass and Biodiesel Content of *Bacillus subtilis* Cultured for 8 Days Under Conditions Similar to *Chlorella* Growth.

Duration of cultivation	0	2	4	6	8
Biomass	11.537 ± 0.870	17.725 ± 1.393	25.298 ± 1.594	27.858 ± 0.838	28.534 ± 0.966
Biodiesel	1.543 ± 0.168	1.925 ± 0.617	2.942 ± 0.099	3.309 ± 0.369	3.516 ± 0.195

Notes: The Bacillus subtilis solution has been pre cultured for 36 hours before being cultured under the same conditions as the growth of Chlorella vulgaris. Values represent the mean ± standard deviation at each time point.

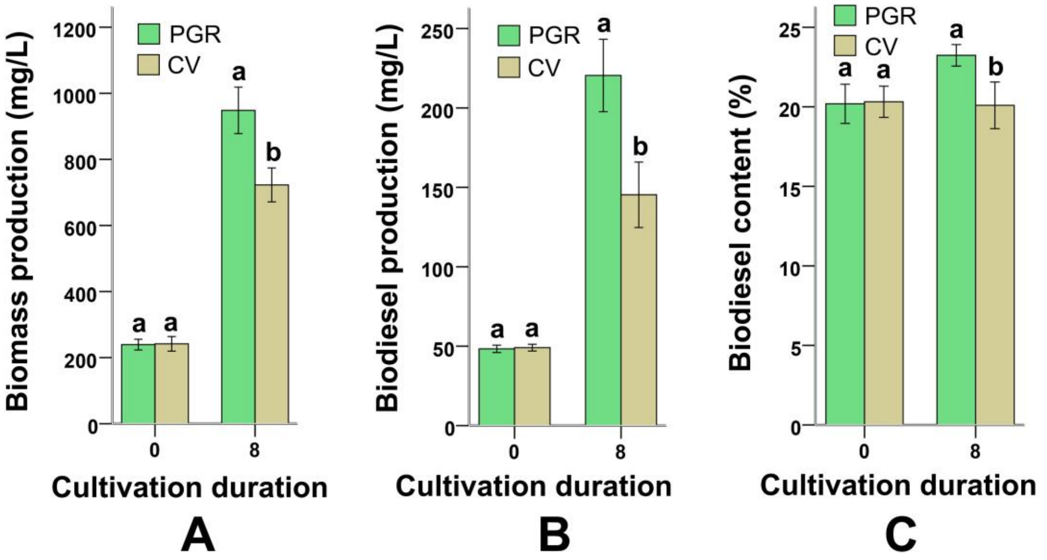

Figure S1. Influence of externally applied plant growth regulators on biomass yield, biodiesel yield, and biodiesel content of Chlorella vulgaris. (A) Biomass production in response to exogenous hormone supplementation. (B) Biodiesel production following exogenous hormone treatment. (C) Biodiesel content under exogenous hormone treatment. The final concentrations of the added plant growth regulators in municipal wastewater were adjusted to levels comparable to those of auxin and gibberellins detected in wastewater after 36 h treatment with Bacillus subtilis. CV: C. vulgaris cultivated directly in municipal wastewater. PGR: C. vulgaris grown in municipal wastewater supplemented with exogenous plant growth regulators.
